# Reduction of Glyoxalase 1 Expression Links Fetal Methylmercury Exposure to Autism Spectrum Disorder Pathogenesis

**DOI:** 10.3390/toxics12070449

**Published:** 2024-06-22

**Authors:** Joseph Wai-Hin Leung, Allison Loan, Yilin Xu, Guang Yang, Jing Wang, Hing Man Chan

**Affiliations:** 1 Regenerative Medicine Program, Ottawa Hospital Research Institute, Ottawa, ON K1H 8L6, Canada; joseph.leung@sickkids.ca (J.W.-H.L.); aloan017@uottawa.ca (A.L.); yxudotcom@gmail.com (Y.X.); 2 Department of Biology, Faculty of Science, University of Ottawa, Ottawa, ON K1H 8M5, Canada; 3 Department of Medical Genetics, Department of Biochemistry and Molecular Biology, Cumming School of Medicine, University of Calgary, Calgary, AB T2N 4N1, Canada; guang.yang2@ucalgary.ca; 4 Alberta Childrens’ Hospital Research Institute, University of Calgary, Calgary, AB T2N 1N4, Canada; 5 Hotchkiss Brain Institute, University of Calgary, Calgary, AB T2N 1N4, Canada; 6 Department of Cellular and Molecular Medicine, Faculty of Medicine, University of Ottawa Brain and Mind Research Institute, University of Ottawa, Ottawa, ON K1H 8M5, Canada

**Keywords:** MeHg, embryonic cortex, radial glia precursors, neuronal differentiation, Glo1, CREB

## Abstract

Glyoxalase 1 (Glo1) is an essential enzyme to detoxify methylglyoxal (MGO), a cytotoxic byproduct of glycolysis. Accumulating studies have shown an important role of Glo1 in regulating cortical development and neurogenesis, potentially contributing to the pathogenesis of autism spectrum disorder (ASD) when impaired. We have previously shown that prenatal exposure to non-apoptotic low-dose methylmercury (MeHg), an environmental pollutant, induces premature cortical neurogenesis and ASD-like behaviors in a rodent model. In this study, we aimed to determine the underlying molecular mechanisms that mediate prenatal MeHg-induced premature neuronal differentiation and abnormal neurodevelopment. Using single-cell RNA sequencing (scRNA-seq) and real-time quantitative PCR (RT-qPCR), we found that prenatal MeHg exposure at a non-apoptotic dose significantly reduced *Glo1* gene expression in embryonic cultured radial glia precursors (RGPs). In cultured RGPs, the knockdown of *Glo1* expression increased neuronal production at the expense of the cultured RGPs population, while overexpression of *Glo1* restored MeHg-induced neuronal differentiation back to normal levels. Furthermore, we found that co-treatment with both MeHg and multiple MGO scavengers or a CREB inhibitor (iCREB) mitigated MeHg-induced premature neuronal differentiation, reinforcing the role of Glo1 and CREB in mediating MeHg-induced neuronal differentiation. Our findings demonstrate a direct link between MeHg exposure and expression of an ASD risk gene *Glo1* in cortical development, supporting the important role of gene–environment interaction in contributing to the etiology of neural developmental disorders, such as ASD.

## 1. Introduction

Glyoxalase 1 (Glo1) is a pivotal enzyme that detoxifies methylglyoxal (MGO), a cytotoxic metabolite that can originate as an endogenous byproduct of glycolysis as well as from other sources such as dietary intake and environmental exposure to pollutants. Emerging research discloses the relationship between Glo1 and autism spectrum disorder (ASD). One study reported that post-mortem brain tissue from patients with ASD had reduced Glo1 enzymatic activity and increased MGO levels compared to control patients [1]. Following this report, several studies have identified genetic variants in the *Glo1* gene and suggest a link between reduced Glo1 enzymatic activity to the etiology of ASD [2,3]. Moreover, an increasing number of studies have focused on Glo1 function in regulating neurodevelopment and neurogenesis. A recent study revealed that Glo1 knockdown leading to MGO accumulation can induce premature neuronal differentiation from embryonic cultured radial glia precursors (RGPs) during cortical development [4]. Another study shows that Glo1 inhibition/MGO accumulation can activate tyrosine receptor kinase B (TrkB) signaling, which in turn stimulates a kinase cascade: phosphorylation of protein kinase B (Akt) leading to phosphorylation of extracellular signal-regulated kinases (ERKs) to enhance cAMP response element-binding protein (CREB) phosphorylation/activation and the expression of the brain-derived neurotrophic factor (BDNF), an integral pathway in neurogenesis [5]. This suggests the Glo1 and CREB may participate in a communal pathway that affects cultured embryonic RGPs’ neuronal differentiation. While the development of the brain is known to be influenced by the environment it is exposed to, the specific mechanisms through which environmental factors may regulate *Glo1* expression remain unclear.

Methylmercury (MeHg) is a well-known environmental toxicant that can pass through the blood–brain barrier and placenta and affect fetal neurodevelopment, causing cognitive deficits and motor dysfunction in children [6,7,8]. MeHg affects millions of people worldwide and is considered one of the top 10 chemicals of major public health concern by the World Health Organization [9]. Our recent study investigated the effect of prenatal non-apoptotic low-dose MeHg exposure during gestation on neurobehavioral outcomes. In this study, we treated pregnant mice with 0 or 0.2 ppm MeHg drinking water from embryonic day 0 (E0) until postnatal day 0 (P0). We found that low-dose MeHg could lead to ASD-like behaviors in adult rodents. This was characterized by impaired communication, sociability, and repetitive behaviors. Moreover, we found that prenatal low-dose MeHg exposure resulted in premature neuronal differentiation during the development of the cerebral cortex [10]. These findings suggest that MeHg, when given at a non-apoptotic dose in vivo, perturbs cortical neurogenesis in the fetal period, leading to long-lasting impacts on neuro-performance. On the other hand, the epidemiological evidence for the relationship between MeHg exposure and ASD remains inconclusive [11,12,13,14,15], and the underlying mechanism between prenatal MeHg exposure and postnatal ASD onset is unknown. Therefore, deciphering the underlying cellular and molecular mechanisms that mediate MeHg-induced abnormal neurodevelopment (premature neuronal differentiation) will provide new insights into how non-genetic factors such as environmental chemical exposure contribute to ASD etiology. 

The goal of this study is to understand the environment–gene interaction in relation to ASD, inspiring the possible biomarkers for the early detection of ASD at high risk and potential targeted therapeutic strategies. We hypothesized that prenatal exposure to MeHg at the non-apoptotic dosage induces neuronal differentiation by reducing the expression of Glo1. Here, we show that the knockdown of Glo1 expression in cultured embryonic RGPs leads to premature neuronal differentiation, and phenocopying MeHg exposure. In contrast, Glo1 overexpression reverses MeHg-induced premature neuronal differentiation back to normal levels. Moreover, the co-treatment of MeHg with either MGO scavengers or a CREB inhibitor in cultured embryonic cortical RGPs could restore MeHg-induced neuronal differentiation. Our study reveals a novel mechanistic link between Glo1 regulation and MeHg-induced adverse effects on brain development.

## 2. Materials and Methods

### 2.1. Single-Cell RNA Sequencing (scRNA-seq)

All scRNA-seq pre-processing was previously described in *iScience,* by Loan et al., 2023 [10]. 

### 2.2. Differential Gene Expression

Differentially expressed genes between control (0 ppm) radial glial precursors (RGPs) (including RGP1 and RGP2 clusters), and MeHg (0.2 ppm) RGPs were identified via the “FindMarkers” function in Seurat v5.0.1 with default settings. Volcano plots were generated using the EnhancedVolcano function from genes identified via “FindMarkers”. Discriminated genes were based on *p*-value adjusted and Log2 fold-change. Log2 fold-change > 0.5 and *p*-value adjusted < 10e^−14^. These differentially expressed genes were used in iRegulon (Cytoscape) to identify gene regulatory networks that connect transcription factors to their predicted target genes as described previously [16].

### 2.3. Primary Cultured RGPs

Primary cultured RGPs were obtained from E11–12 pooled cortices dissected from CD-1 mice (Charles River Laboratories) as previously described [17]. Briefly, embryos were transferred to ice-cold Hanks’ balanced salt solution (HBSS) (cat#14175103, Thermo Fisher Scientific, Waltham, MA, USA), and the cerebral cortices were isolated from the brain after the meninges were removed. The cortical tissue was mechanically triturated with a plastic pipette and seeded on coverslips in a 24-well plate or directly into a 6-well plate (Thermo Fisher Scientific), both pre-coated with 15% poly-L-ornithine (PLO) (cat#72302, Sigma-Aldrich, Burlington, MA, USA) and 5% laminin (cat#CB40232, Thermo Fisher Scientific). 

For immunocytochemical experiments, cells were plated in a 24-well plate at a density of 200,000 cells/mL. For RT-qPCR experiments, 1,000,000 cells were seeded in each well of a 6-well plate. The cultured RGPs were cultured in a Neurobasal Medium (cat#21103049, Thermo Fisher Scientific, containing 4500 mg/L glucose) containing 1X GlutaMAX supplement (cat#35050061, Thermo Fisher Scientific), 2% B27 supplement (cat#17504044, Thermo Fisher Scientific), 1% penicillin-streptomycin (cat#15140122, Thermo Fisher Scientific), and 40 ng/mL fibroblast growth factor 2 (FGF2) (cat#10018B, PeproTech, Cranbury, NJ, USA). 

For the shRNA experiment (*Glo1* knockdown), cultured RGPs were cultured in Neurobasal Medium (cat#A2477501, Thermo Fisher Scientific) with a reduced glucose (cat#G7021, Sigma-Aldrich) concentration (2250 mg/L). Since Glo1 plays an important role in detoxifying the toxic MGO, knocked down *Glo1* can lead to an increase in MGO concentration, resulting in oxidative stress and cell death [18]. To minimize the toxic effect of MGO, we limited the supply of glucose in the medium to reduce the MGO accumulation. We found that a medium with a glucose concentration of 2250 mg/L is optimal for the shRNA knockdown experiment.

### 2.4. Pharmacological Treatments

#### 2.4.1. 250 nM MeHg

Primary E11–12 cultured RGPs were exposed to 0 nM or 250 nM MeHg [17] for 24 h or 48 h. A concentration of 250 nM MeHg was achieved by a 1:250 dilution of 62.5 µM MeHg that was freshly prepared from a stock solution of 4 mM MeHg(II)Cl (cat#33553, Alfa Aesar, Ward Hill, MA, USA) through serial dilution with the culture medium.

#### 2.4.2. Aminoguanidine (AG)

Cultured RGPs were exposed to 0 nM or 100 µM AG (cat#396494, Sigma-Aldrich) for 48 h. A concentration of 100 µM AG was prepared by initially diluting it in 100% DMSO to a concentration of 100 mM and then further diluting it in a culture medium 1000 times to achieve the final concentration of 100 µM AG, with a final DMSO concentration of 0.1%. For the MeHg + AG co-treatment experiment, experimental conditions were (i) Control (0 nM MeHg + DMSO), (ii) 250 nM MeHg + DMSO, (iii) 100 µM AG, (iv) 250 nM MeHg + 100 µM AG.

#### 2.4.3. N-acetyl-l-cysteine (NAC) 

Primary cultured cells were exposed to 0 nM or 600 µM NAC (cat#A9165, Sigma-Aldrich) for 48 h. A concentration of 600 µM NAC was achieved by diluting it in 100% DMSO to 600 mM and then further diluting the solution 1000 times in a culture medium. This resulted in a final concentration of 600 µM NAC, with a DMSO concentration of 0.1%. For the MeHg + NAC co-treatment experiment, the experimental conditions were (i) control (0 nM MeHg + DMSO), (ii) 250 nM MeHg + DMSO, (iii) 600 µM NAC, (iv) 250 nM MeHg + 600 µM NAC.

#### 2.4.4. CREB Inhibitor (iCREB)

Primary cultured cells were exposed to 0 nM or 80 nM iCREB, 666-15 (cat#30780, Cayman Chemical Company, Ann Arbor, MI, USA) for 48 h. A concentration of 80 nM iCREB was achieved by diluting it in 100% DMSO to 80 µM and then further diluting it in a culture medium 1000 times. This resulted in a final concentration of 80 nM iCREB, with a DMSO concentration of 0.1%. A concentration of 80 nM iCREB was achieved by diluting iCREB in DMSO. For MeHg + iCREB co-treatment experiment conditions were (i) control (0 nM MeHg + DMSO), (ii) 250 nM MeHg + DMSO, (iii) 80 nM iCREB, (iv) 250 nM MeHg + 80 nM iCREB.

#### 2.4.5. Plasmid Transfections

For the cultured RGPs transfections, 0.9 μg DNA (1:2 ratio of *PB-CAG-eGFP* versus the *pSUPER-Glo1 shRNA* or an empty vector (EV); 1:2 ratio of *PB-CAG-eGFP* versus *pcDNA3-Flag-Glo1* or EV) and 1 μL Lipofectamine™ Stem Transfection Reagent (Thermo Scientific STEM00003) were mixed with 50 μL Opti-MEM medium, incubated for 30 min and added to cultured RGPs 24 h after plating. The knockdown efficiency of *Glo1*-shRNA has been validated in a previous publication both in culture and in vivo [4]. The expression efficiency of the Flag-*Glo1* plasmid has been validated in a previous publication as well [4].

### 2.5. Reverse Transcription-Quantitative Real-Time Polymerase Chain Reaction (RT-qPCR)

Cultured RGPs were cultured as previously described. RNA was extracted from cultured RGPs using the PureLink RNA Mini Kit (cat#12183020, Thermo Fisher). Complementary DNA (cDNA) was synthesized using a QuantiTect Reverse Transcription Kit (cat#205311, Qiagen, Hilden, Germany). The RT-qPCR was performed with a SensiFAST SYBR Lo-ROX Kit (cat# BIO-94005, Bioline, Alvinston, Ontario, Canada) on an Mx3000P qPCR System (Agilent, Santa Clara, CA, USA). All qPCRs were performed using the same protocol (95 °C for 2 min for 1 cycle; 95 °C for 10 s, 58 °C, 15 s; 72 °C, 20 s for 40 cycles). All qPCR samples were performed in technical duplicates and then averaged. *Glyceraldehyde-3-phosphate dehydrogenase* (*GAPDH*) was used as a loading control and the fold expression normalized to *GAPDH* was used as a readout. PCR primer sequences: *Glo1*-forward: 5′-GATTTGGTCACATTGGGATTGC-3′, *Glo1*-reverse: 5′-TCCTTTCATTTTCCCGTCATCAG-3′, *GAPDH*-forward: 5′-AGGTCGGTGTAACGGATT-3′, *GAPDH*-reverse: 5′-TGTAGACCATGTAGTTGAG-3′. Primers were validated by running gel electrophoresis and experimental conditions were optimized. Data were analyzed using AriaMX (Agilent, Santa Clara, CA, USA).

### 2.6. Immunocytochemistry

Cultured RGPs were cultured as previously described. Cells were fixed in 4% paraformaldehyde for 10 min after 48 h in culture and then blocked with 10% normal goat serum (NGS) (cat#16050122, Thermo Fisher Scientific) diluted in 1× PBS with 0.3% Triton X-100 (PBST). The cells were incubated with primary antibodies diluted in 10% NGS in PBS with 0.3% Triton X-100 and then incubated in a humid chamber at 4 °C overnight. Following this, cells were incubated with secondary antibodies diluted in PBST for 1 h at room temperature. After rinsing with PBS, the coverslips were mounted in a Lab Vision PermaFluor Aqueous Mounting Medium (cat#TA-030-FM, Thermo Fisher Scientific). The culture was washed three times for 5 min/time with 1× PBS between each step.

The primary antibodies used for immunocytochemistry were mouse anti-βIII-tubulin (cat#801201, BioLegend, San Diego, California, USA, 1:1000), rabbit anti-Pax6 (cat#901301, BioLegend, 1:1000), rabbit anti-Sox2 (cat#AB5603MI, Sigma-Aldrich, 1:500), and mouse anti-Ki67 (cat#ab15580, Abcam, Cambridge, UK, 1:500). The secondary antibodies used were donkey anti-rabbit Alexa Fluor 555 (cat#A31572, Thermo Fisher Scientific, 1:500) and goat anti-mouse Alexa Fluor 488 (cat#A32723, Thermo Fisher Scientific, 1:500). Nuclear counterstaining was performed with Hoechst 33342 (cat#4082, Cell Signalling Technology, Danvers, MA, USA, 1:1000).

Digital image acquisition was performed using Zeiss Imager M.1 fluorescent microscopy with Zeiss Axiovision software containing z-axis capability (Carl Zeiss Microscopy, Thornwood, NY, USA). For *Glo1* overexpression and knockdown experiments, at least 200 successfully transfected cells (GFP^+^), chosen from random microscopic fields, were examined. The percentage of GFP^+^ cells expressing the markers of interest was studied. In the remaining experiments, five random images (20× magnification) per condition were captured for quantitative analysis. At least three independent experiments from three pregnant mice were conducted for all conditions. Quantification was performed using Image J.

## 3. Results

*Glo1* expression is reduced in radial glial precursors (RGPs) following prenatal low-dose MeHg treatment.

**Figure 1:**
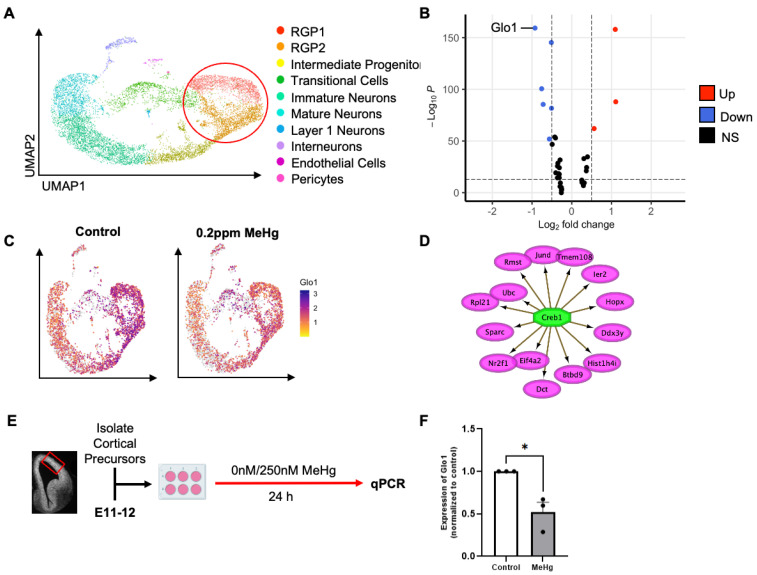
*Glo1* expression is reduced in radial glial precursors (RGPs) following prenatal low-dose methylmercury (MeHg) treatment. (**A**) Visualization of cells from control (0 ppm) and MeHg (0.2 ppm) treated cortical tissue, colored by Seurat clustering and annotated by cell type, red circle represents cell populations (RGP1 and RGP2) used for downstream differentially expressed gene (DEG) analysis (**B**) Volcano plot of differentially expressed genes between control (0 ppm) RGP1 and RGP2 and MeHg (0.2 ppm) RGP1 and RGP2. Discriminated based on *p*-value adjusted and log2 fold-change. Log2 fold-change > 0.5 and *p*-value adjusted < 10e^−14^. (**C**) Visualization of the total cell population after PCA and UMAP, colored by expression of *Glo1*. (**D**) Transcription factor CREB1 was identified from iRegulon (Cytoscape) and its direct transcriptional targets. (**E**) Experimental timeline following radial glia precursor (RGP) isolation from embryonic day 11–12 (E11–12) CD1 mice, created with BioRender.com. The red box indicates the region dissected to obtain RGPs. (**F**) Cells were exposed to two conditions: (i) control (0 nM MeHg) and (ii) 250 nM MeHg for 24 h, at which point they were lysed. Quantitative analysis of *Glo1* expression, over GAPDH, normalized to control. n = 4 independent experiments, Student *t*-test, * *p* < 0.05. Error bars indicate the SEM.

Using single-cell RNA sequencing (scRNA-seq) analysis in our recently published work [10], we found that prenatal non-apoptotic low-dose 0.2 ppm MeHg exposure favors embryonic radial glial precursor 1 (RGP1) to directly differentiate into cortical neurons, omitting the intermediate progenitor stage (Ref. [10], Figure 1A). Following this, we performed downstream analysis to probe differentially expressed genes (DEGs), specifically in the RGP1 and RGP2 populations using the same scRNA-seq dataset (Table 1). This analysis revealed a constant reduction in the expression of the *Glo1* gene across different cell clusters (Figure 1B). Consistently, *Glo1* was identified as the most statistically significant downregulated gene in the volcano plot (Figure 1C). Further analysis using iRegulon [16] identified CREB1 as a top candidate transcription factor in RGPs to directly regulate the expression of the 14 DEGs, but *Glo1* was not the direct target gene (Figure 1D). This suggests that Glo1-controlled CREB activity, supported by the previous work [5], may mediate MeHg-induced embryonic RGP neuronal differentiation. 

To assess the role of Glo1 in MeHg-induced neuronal differentiation, we used a monolayer embryonic RGPs culture model (Figure 1E) and exposed precursor cells to low-dose MeHg (Figure 1F). In line with our in vivo model, reverse transcription quantitative real-time polymerase chain reaction (RT-qPCR) analysis showed a reduction in *Glo1* expression in RGP cultures in response to MeHg exposure.

*Glo1* reduction facilitates premature neuronal differentiation in culture.

**Figure 2:**
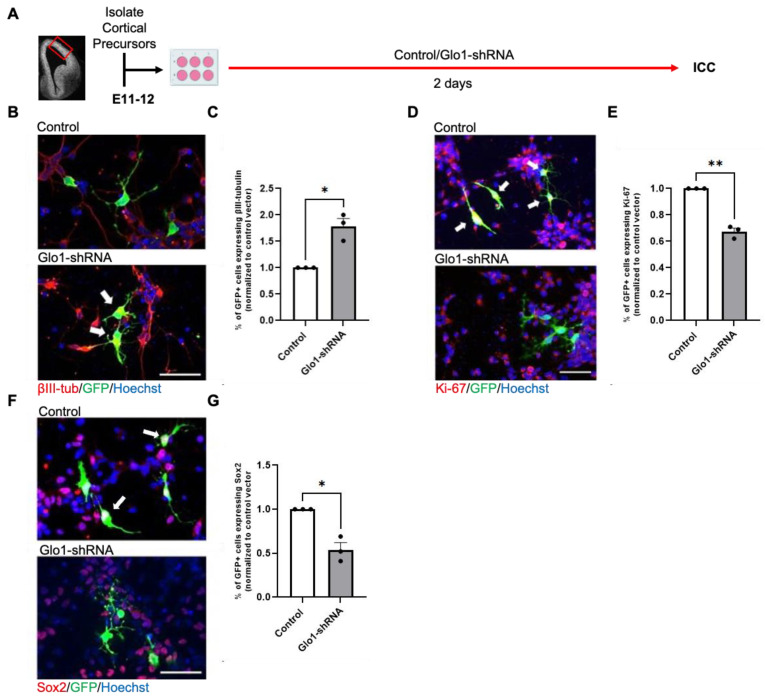
Glo1 reduction promotes premature neuronal differentiation in culture. (**A**) Workflow for culturing embryonic day 11–12 (E11–12) RGPs, created with BioRender.com. The red box indicates the region dissected to obtain RGPs. (**B**,**D**,**F**) Images of RGPs that were transfected with GFP reporter construct, together with Control or *Glo1*-shRNA. GFP (green), βIII-tubulin (**B**, red), Ki-67 (**D**, red), or Sox2 (**F**, red) and counterstained for Hoechst (blue). White arrows indicated (**B**) βIII-tubulin^+^/GFP^+^ cells, (**D)** Ki-67^+^/GFP^+^ cells, and (**F**) Sox2^+^/GFP^+^ cells. Scale bar: 50 μm. (**C**,**E**,**G**) Quantitative analysis of the percentage of GFP^+^ cells expressing βIII-tubulin^+^ (**C**), Ki-67^+^ (**E**), and Sox2^+^ (**G**), over total GFP^+^ cells, normalized to a control group. n = 3 independent experiments, Student *t*-test, * *p* < 0.05, ** *p* < 0.01. Error bars indicate the SEM.

To assess the effect of Glo1 downregulation on cultured embryonic RGPs, we transfected E11-E12 embryonic RGPs with a GFP reporter construct, together with a validated *Glo1*-shRNA [4]. After 2 days in culture following transfections, immunocytochemical analysis revealed that knockdown of *Glo1* increased the percentage of βIII tubulin^+^ neurons (Figure 2B,C) but reduced the number of Ki-67^+^ proliferating precursors (Figure 2D,E) and the number of Sox2^+^ RGPs (Figure 2F,G), phenocopying MeHg-induced neuronal differentiation in culture [17].

*Glo1* overexpression reduces MeHg-induce premature neuronal differentiation.

**Figure 3:**
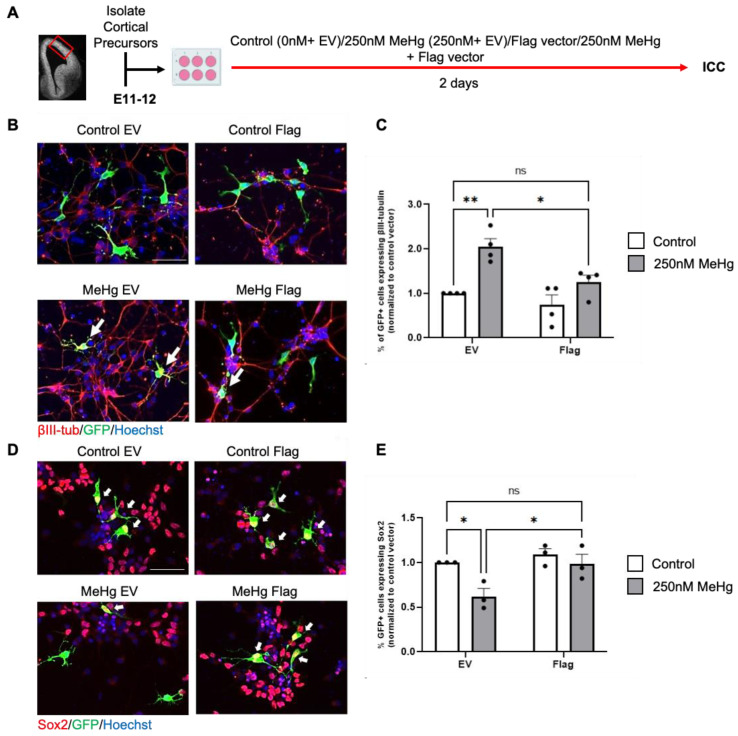
*Glo1* overexpression restores MeHg-induced premature neuronal differentiation. (**A**) Workflow for culturing E11–12 RGPs, created with BioRender.com. The red box indicates the region dissected to obtain RGPs. (**B**,**D**) Images of RGPs treated with (i) control (0 nM MeHg + GFP construct with EV plasmids), (ii) 250 nM MeHg + GFP construct with EV plasmids, (iii) 250 nM MeHg + GFP construct with Flag-*Glo1* plasmids, and or iv) 250 nM MeHg + GFP construct with Flag-*Glo1*. GFP (green), βIII-tubulin (**B**, red) or Sox2 (**D**, red), and Hoechst (blue). White arrows indicated (**B**) βIII-tubulin^+^/GFP^+^ cells and (**D**) Sox2^+^/GFP^+^ cells. Scale bar: 50 μm. (**C**) Quantitative analysis of the percentage of GFP^+^/βIII-tubulin^+^ cells over total GFP^+^ cells, normalized to a control/EV, group, n = 4 independent experiments, Two-way ANOVA (transfection × Hg interaction F(1, 12) = 2.866, P = 0.1163, transfection F(1, 12) = 10.68, P = 0.0067, Hg F(1, 12) = 23.62, P = 0.0004). (**E**) Quantitative analysis of the percentage of GFP^+^/Sox2^+^ cells over total GFP^+^ cells, normalized to a control/EV group, n = 3 independent experiments, Two-way ANOVA (transfection × Hg interaction F(1, 8) = 3.147, P = 0.1140, transfection F(1, 8) = 9.605, P = 0.0147, Hg F(1, 8) = 8.326, P = 0.0203). post-hoc, * *p* < 0.05; ** *p* < 0.01. Error bars indicate the SEM.

To investigate if overexpression of Glo1 can block premature neuronal differentiation caused by 250 nM MeHg exposure, we used our embryonic RGP cultures treated with either (i) control (0 nM MeHg + GFP construct with empty vector (EV) plasmids), (ii) 250 nM MeHg + GFP construct with EV plasmids, (iii) 250 nM MeHg + GFP construct with Flag-*Glo1* plasmids, and (iv) 250 nM MeHg + GFP construct with Flag-*Glo1* plasmids for 2 days in culture (Figure 3A). Immunocytochemical analysis showed that overexpression of *Glo1* can reverse the increased number of βIII tubulin^+^ neurons (Figure 3B,C) and reduced number of Sox2^+^ RGPs (Figure 3D,E) caused by 250 nM MeHg back to the normal level. 

MGO-regulated CREB pathway mediates MeHg-induced neuronal differentiation. 

**Figure 4:**
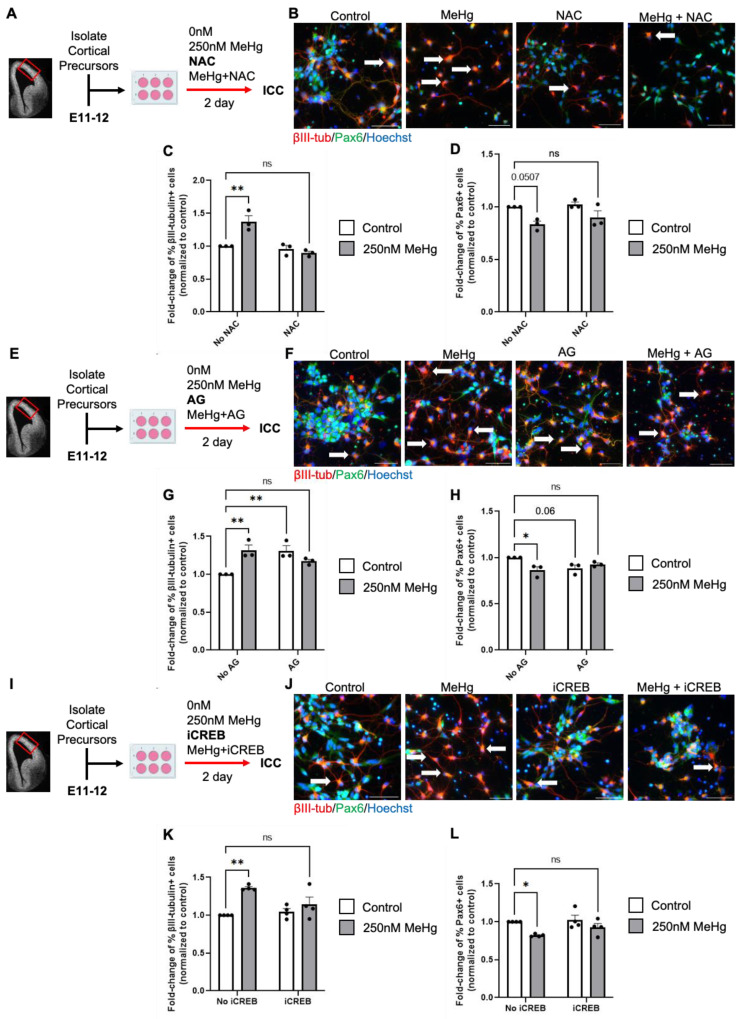
Methylglyoxal (MGO) and the Akt-ERK-CREB pathway mediates MeHg-induced neuronal differentiation. (**A**) Workflow of E11–12 RGPs exposed to four conditions: (i) control (0 ppm MeHg + 0 μM NAC), (ii) 250 nM MeHg + 0 μM NAC, (iii) 0 nM MeHg + 600 μM NAC, (iv) co-treatment of 250 nM MeHg + 600 μM NAC for 2 days, followed by immunocytochemical analysis. The red box indicates the region dissected to obtain RGPs. (**B**) Images of RGPs immunostained for Pax6 (green), βIII-tubulin (red), and counterstained for Hoechst (blue). White arrows indicated βIII-tubulin^+^ cells. Scale bar: 50 μm. (**C**) Quantitative analysis of βIII-tubulin^+^ cells, normalized to a control group, n = 3 independent experiments, Two-way ANOVA (NAC × MeHg interaction F(1, 8) = 17.25, P = 0.0032, NAC F(1, 8) = 24.11, P = 0.0012, MeHg F(1, 8) = 8.542, P = 0.0192). (**D**) Quantitative analysis of Pax6^+^ cells, normalized to a control group, n = 3 independent experiments, Two-way ANOVA (NAC × MeHg interaction F(1, 8) = 0.3460, P = 0.5726, NAC F(1, 8) = 1.473, P = 0.2596, MeHg F(1, 8) = 15.41, P = 0.0044). (**E**) Workflow of RGPs exposed to four conditions: (i) control (0 nM MeHg + 0 μM AG), (ii) 250 nM MeHg + 0 μM AG, (iii) 0 nM MeHg + 100 μM AG, (iv) co-treatment of 250 nM MeHg + 100 μM AG for 2 days, followed by immunocytochemical analysis. The red box indicates the region dissected to obtain RGPs. (**F**) Images of RGPs immunostained for Pax6 (green), βIII-tubulin (red), and counterstained for Hoechst (blue). White arrows indicated βIII-tubulin^+^ cells. Scale bar: 50 μm. (**G**) Quantitative analysis of βIII-tubulin^+^ cells, normalized to a control group, n = 3 independent experiments, Two-way ANOVA (AG × MeHg interaction F(1, 8) = 21.54, P = 0.0017, AG F(1, 8) = 2.756, P = 0.1355, MeHg F(1, 8) = 3.258, P = 0.1087. (**H**) Quantitative analysis of Pax6^+^ cells, normalized to control, n = 3 independent experiments, Two-way ANOVA (AG × MeHg interaction F(1, 8) = 11.05, P = 0.0105 AG F(1, 8) = 0.8509, P = 0.3833, MeHg F(1, 8) = 3.127, P = 0.1150). (**I**) Workflow of RGPs exposed to four conditions: (i) control (0 nM MeHg + 0 nM iCREB, (ii) 250 nM MeHg + 0 nM iCREB, (iii) 80 nM iCREB + 0 nM MeHg, (iv) co-treatment 250 nM MeHg + 80 nM iCREB for 2 days, followed by immunocytochemical analysis. The red box indicates the region dissected to obtain RGPs. (**J**) Images of RGPs immunostained for Pax6 (green), βIII-tubulin (red), and counterstained for Hoechst (blue). White arrows indicated βIII-tubulin^+^ cells. Scale bar: 50 μm. (**K**) Quantitative analysis of βIII-tubulin^+^ cells, normalized to control, n = 4 independent experiments, Two-way ANOVA (iCREB × MeHg interaction F(1, 12) = 6.012 P = 0.0305, iCREB F(1, 12) = 2.554, P = 0.1360, MeHg F(1, 12) = 18.17, P = 0.0011. (**L**) Quantitative analysis of Pax6^+^ cells, normalized to control, n = 4 independent experiments, Two-way ANOVA (iCREB × MeHg interaction F (1, 12) = 1.057, P = 0.3242, iCREB F(1, 12) = 2.633, P = 0.1306, MeHg F(1, 12) = 11.73, P = 0.005. post hoc, * *p* < 0.05; ** *p* < 0.01. Error bars indicate the SEM.

To test whether the accumulated MGO due to reduced *Glo1* expression is responsible for MeHg-induced premature neuronal differentiation, we cultured embryonic RGPs in the presence of MeHg (250 nM) and MGO scavengers, N-acetyl-l-cysteine (NAC) or aminoguanidine (AG). First, E11–12 RGPs were treated with either (i) control (0 nM MeHg + 0 nM NAC), (ii) 250 nM MeHg, (iii) 600 µM NAC, or (iv) 250 nM MeHg + 600 µM NAC for 2 days in culture (Figure 4A). Immunocytochemical analysis revealed that NAC co-treatment with MeHg prevented MeHg-induced premature neuronal differentiation by recovering the increased number of β-III tubulin^+^ neurons and reducing the number of Pax6^+^ RGPs back to the normal level (Figure 4B–D). Subsequently, we used a second MGO scavenger, 100 µM AG, and repeated the aforementioned experiment (Figure 4E). Immunocytochemical analysis revealed that co-treatment of AG with MeHg can also prevent MeHg-induced premature neuronal differentiation as NAC did (Figure 4F–H). However, AG treatment alone increased the number of β III tubulin^+^ neurons and reduced the number of Pax6^+^ RGPs. Interestingly, AG is similar in structure to metformin [19], an FDA-approved drug we have previously shown to cause premature neuronal differentiation in cultured E11–12 RGPs [20]. 

Since our previous work showed that MeHg promoted CREB phosphorylation at Ser133, to induce premature neuronal differentiation, we employed CREB inhibitor (666-15), in the cultured embryonic RGPs: (i) control (0 nM MeHg + 0 nM iCREB), (ii) 250 nM MeHg + 0 nM iCREB, (iii) 0 nM MeHg + 80 nM iCREB, or (iv) 250 nM MeHg + 80 nM iCREB for 2 days in culture (Figure 4I). We showed that 80 nM iCREB can recover MeHg-induced premature neuronal differentiation as MGO scavengers did (Figure 4J–L). Since previous work reported that accumulated MGO can stimulate CREB activity by promoting CREB phosphorylation at Ser133 [5], our work, here, suggests that the MGO-regulated CREB pathway mediates MeHg-induced neuronal differentiation. 

## 4. Discussion

Our previous publication found that exposure to the environmental contaminant MeHg during gestation could lead to ASD-like behaviors in adult rodents and premature neuronal differentiation of the cerebral cortex [10]. The present study demonstrates how MeHg interacts with gene expression to impact neural developmental processes, potentially contributing to the onset of previously observed ASD symptoms. Specifically, our study reports four major findings. First, we reveal that prenatal exposure to non-apoptotic MeHg significantly reduces *Glo1* gene expression in embryonic RGPs both in vivo and in culture. Second, the knockdown of *Glo1* expression in embryonic RGPs can cause premature neuronal differentiation, phenocopying low-dose MeHg exposure. Third, *Glo1* overexpression in embryonic RGPs prevents MeHg-induced premature neuronal differentiation. Finally, co-treatment of MeHg with either MGO scavengers or iCREB in embryonic RGPs reverses MeHg-induced neuronal differentiation back to normal. 

An increasing number of studies have shown that reduced Glo1 enzyme activity due to *Glo1* polymorphisms and increased MGO levels are found in post-mortem brain tissues from patients with ASD, potentially contributing to the etiology of ASD [2,3]. Intriguingly, gestational diabetes often causes an overproduction of MGO, a circulating toxic intermediate metabolite that can pass through the placenta barrier to enter the fetal circulation [4,21,22]. At the same time, gestational diabetes has been associated with neurodevelopmental disorders, including ASD [23,24,25]. In this regard, the Glo1-regulated MGO pathway seems to be a pivotal node that can connect environmental factors to neural developmental disorders, such as ASD. In this study, we show that fetal exposure to non-apoptotic MeHg causes a reduction in *Glo1* expression, leading to an accumulation of MGO, which may underlie MeHg-induced premature neuronal differentiation of embryonic RGPs. Our recently published work also shows that the same dosage of MeHg in embryos can lead to ASD-like behaviors in adult rodents [10]. This suggests that the gene–environment interaction between MeHg and *Glo1* gene expression may contribute to ASD pathogenesis by perturbing the embryonic neural precursor development. Aligning with our findings, recent work has shown that the *Glo1*-regulated MGO pathway is important to regulating embryonic neural precursor maintenance, and perturbations in this pathway in vivo can lead to premature neuronal differentiation during the embryonic stage and long-lasting alterations in adult neural precursor pools [4]. It is important to note that a recent publication studied the effect of prenatal exposure to valproic acid (VPA) on Glo1 levels in postnatal mice. Here, the researchers found that VPA exposure during gestation results in increased Glo1 levels starting at 8 weeks postnatally which are accompanied by ASD-like behaviors [26]. It is interesting to observe that two different environmental factors can cause different outcomes of Glo1 expression, in relation to ASD progression. Our work shows that prenatal MeHg treatment causes an immediate reduction in Glo1 to impact the cortical development, potentially leading to ASD behavior later in life. On the other hand, prenatal VPA exposure leads to delayed Glo1 accumulation postnatally to alter the neuronal circuitry, contributing to ASD etiology. Together, these findings suggest that Glo1 is an important player contributing to non-genetic ASD.

Previous work from our lab put forward the theory that CREB phosphorylation is essential for low-dose MeHg to promote neuronal differentiation. Our current results suggest that CREB activation may act as downstream signaling of reduced *Glo1* caused by MeHg exposure. This postulation is supported by a recent discovery that MGO accumulation due to reduced Glo1 levels can act on tyrosine kinase receptors to stimulate the Akt signaling pathway to enhance CREB phosphorylation/activity [5]. Moreover, the same concentration of MeHg treatment (250 nM) in other cell lines is capable of stimulating Akt signaling to promote CREB phosphorylation/activation [27].

Overall, our study shows that reduced *Glo1* expression is essential for prenatal non-apoptotic low-dose MeHg exposure to induce premature neuronal differentiation in fetal cortical development. Our findings demonstrate a direct link between MeHg exposure and expression of an ASD risk gene Glo1 in cortical development, supporting the important role of gene–environment interaction in contributing to the etiology of neural developmental disorders, such as ASD. Future studies could investigate whether Glo1 can serve as a biomarker to identify a group of infants exposed to MeHg with a high risk of developing an ASD-like phenotype.

## Figures and Tables

**Table 1:** Differentially expressed genes between 0 ppm and 0.2 ppm MeHg-treated RGPs were identified via the FindMarkers function.

*Gene*	*p-Value*	*Average log2FC*	*Regulation (Relative to 0 ppm RGPs)*
*Glo1*	3.08 × 10^−164^	−0.9240032	Downregulated
*Rpl26*	2.85 × 10^−150^	−0.5132826	Downregulated
*Cwc22*	1.48 × 10^−105^	−0.7570934	Downregulated
*Gm47283*	2.01 × 10^−90^	−0.7192588	Downregulated
*Tpm3−rs7*	1.08 × 10^−86^	−0.5086832	Downregulated
*Ddx3y*	7.17 × 10^−59^	−0.4261163	Downregulated
*Eif2s3y*	6.14 × 10^−58^	−0.4087932	Downregulated
*Rsrp1*	7.15 × 10^−57^	−0.5629889	Downregulated
*1810026B05Rik*	8.95 × 10^−52^	−0.4919984	Downregulated
*Actg1*	1.06 × 10^−36^	−0.2934132	Downregulated
*Btbd9*	1.43 × 10^−33^	−0.348239	Downregulated
*1110038B12Rik*	1.04 × 10^−30^	−0.3580768	Downregulated
*Gm21887*	3.84 × 10−^29^	−0.3112397	Downregulated
*Nr2f1*	2.92 × 10^−24^	−0.4025072	Downregulated
*Eif4a2*	2.04 × 10^−23^	−0.3295204	Downregulated
*Pop4*	7.24 × 10^−23^	−0.3122949	Downregulated
*Snhg15*	1.97 × 10^−19^	−0.3127317	Downregulated
*Mt1*	2.23 × 10^−19^	−0.3604426	Downregulated
*Gadd45g*	5.24 × 10^−11^	−0.2945627	Downregulated
*Lix1*	4.93 × 10^−10^	−0.2608959	Downregulated
*Ier2*	3.09 × 10^−08^	−0.2509708	Downregulated
*Sparc*	2.02 × 10^−07^	−0.258431	Downregulated
*Rmst*	0.00454018	−0.2614077	Downregulated
*Xist*	5.78 × 10^−163^	1.09666796	Upregulated
*Rpl21*	6.13 × 10^−93^	1.10845559	Upregulated
*Ubc*	4.95 × 10^−67^	0.56325077	Upregulated
*Tsix*	1.08 × 10^−39^	0.39728863	Upregulated
*Ubb*	6.08 × 10^−38^	0.30566344	Upregulated
*Tmem14c*	2.16 × 10^−29^	0.37480296	Upregulated
*Hist1h3c*	3.85 × 10^−26^	0.36725854	Upregulated
*Jund*	3.74 × 10^−17^	0.25385254	Upregulated
*Polr2k*	4.83 × 10^−15^	0.25872311	Upregulated
*Tmem108*	8.33 × 10^−15^	0.26318465	Upregulated
*Dct*	1.67 × 10^−14^	0.31588436	Upregulated
*Hist1h4i*	3.47 × 10^−13^	0.29253036	Upregulated
*Hopx*	7.64 × 10^−12^	0.29434801	Upregulated

## Data Availability

The data presented in this study are available on request from the corresponding authors.
